# The Effects of Iodine Supplementation in Pregnancy on Iodine Status, Thyroglobulin Levels and Thyroid Function Parameters: Results from a Randomized Controlled Clinical Trial in a Mild-to-Moderate Iodine Deficiency Area

**DOI:** 10.3390/nu11112639

**Published:** 2019-11-04

**Authors:** Simona Censi, Sara Watutantrige-Fernando, Giulia Groccia, Jacopo Manso, Mario Plebani, Diego Faggian, Monica Maria Mion, Roberta Venturini, Alessandra Andrisani, Anna Casaro, Pietro Vita, Alessandra Avogadro, Marta Camilot, Carla Scaroni, Loris Bertazza, Susi Barollo, Caterina Mian

**Affiliations:** 1 Department of Medicine (DIMED), Endocrinology Unit; University of Padova, 35121 Padova, Italy; simona.censi@phd.unipd.it (S.C.); sara.watutantrige@gmail.com (S.W.-F.); giulia.groccia@gmail.com (G.G.); jacopo.manso@gmail.com (J.M.); anna.casaro@aulss6.veneto.it (A.C.); pietro.vida@aulss6.veneto.it (P.V.); alessandra.avogadro@aulss6.veneto.it (A.A.); carla.scaroni@unipd.it (C.S.); loris.bertazza@unipd.it (L.B.); susibarollo@yahoo.it (S.B.); 2 Laboratory Medicine, Department of Medical and Surgical Sciences, University of Padua, 35121 Padua, Italy; mario.plebani@unipd.it (M.P.); diego.faggian@aopd.veneto.it (D.F.); monica.mion@aopd.veneto.it (M.M.M.); roberta.venturini@aopd.veneto.it (R.V.); 3 Department of Women’s and Children’s Health, University of Padua, Salus Pueri, 35128 Padua, Italy; alessandra.andrisani@unipd.it; 4 Department of Pediatrics, Regional Centre for Newborn Screening, Diagnosis and Treatment of Inherited Metabolic Diseases and Congenital Endocrine Diseases, Azienda Ospedaliera Universitaria Integrata, 37134 Verona, Italy; marta.camilot@aovr.veneto.it

**Keywords:** iodine, pregnancy, mild-to-moderate iodine deficiency, randomized and placebo-controlled trial

## Abstract

Background: Iodine supplementation during pregnancy in areas with mild-to-moderate iodine deficiency is still debated. Methods: A single-center, randomized, single-blind and placebo-controlled (3:2) trial was conducted. We enrolled 90 women before 12 weeks of gestation. From enrollment up until 8 weeks after delivery, 52 women were given an iodine supplement (225 ug/day, potassium iodide tablets) and 38 were given placebo. At recruitment (T0), in the second (T1) and third trimesters (T2), and 8 weeks after delivery (T3), we measured participants’ urinary iodine-to-creatinine ratio (UI/Creat), thyroid function parameters (thyroglobulin (Tg), TSH, FT3, and FT4), and thyroid volume (TV). The newborns’ urinary iodine concentrations were evaluated in 16 cases. Results: Median UI/Creat at recruitment was 53.3 ug/g. UI/Creat was significantly higher in supplemented women at T1 and T2. Tg levels were lower at T1 and T2 in women with UI/Creat ≥ 150 ug/g, and in the Iodine group at T2 (*p* = 0.02). There was a negative correlation between Tg and UI/Creat throughout the study (*p* = 0.03, *r* = −0.1268). A lower TSH level was found in the Iodine group at T3 (*p* = 0.001). TV increased by +Δ7.43% in the Iodine group, and by +Δ11.17% in the Placebo group. No differences were found between the newborns’ TSH levels on screening the two groups. Conclusion: Tg proved a good parameter for measuring iodine intake in our placebo-controlled series. Iodine supplementation did not prove harmful to pregnancy in areas of mild-to-moderate iodine deficiency, with no appreciable harmful effect on thyroid function.

## 1. Introduction

According to the World Health Organization (WHO) guidelines, median urinary iodine values ≥150 ug/L are consistent with an adequate iodine intake for pregnant women [1]. The detrimental impact of severe iodine deficiency during pregnancy is well known, since adequate thyroid hormone levels are fundamental to proper neurological development in the fetus [2]. The effect of mild-to-moderate iodine deficiency in pregnancy (urinary iodine concentrations (UIC) between 50 and 150 ug/L) [3] is less clear [4], however, and the benefits of supplementation in this case are less obvious [4]. Mild-to-moderate iodine deficiency during pregnancy has been correlated with an increased thyroid size [5] and thyroid disorders [6] in the mother, as well as an impaired cognitive development in her child, in many but not all studies [7]. A recent survey on 6180 mother-child pairs conducted in areas of mild-to-moderate iodine deficiency found a positive, curvilinear correlation between women’s iodine/creatinine ratios (UI/Creat) before 14 weeks of gestation and their offspring’s mean verbal IQs [8]. The marker most often used to measure iodine status at population level is the median UIC, but it only reflects iodine intake in the previous 24–48 h, and it may be influenced by renal function [9]. A more accurate method would be to consider UI/Creat [10], bearing in mind that glomerular filtration rates are 30% higher in pregnancy [11]. A recent work documented how UI/Creat better reflects 24-h urinary iodine excretion and circulating iodine levels in pregnancy than UIC [12]. Neither UIC nor UI/Creat provide any direct information about thyroid function. Thyroglobulin (Tg) is the precursor of thyroid hormone synthesis and it has proven itself a good marker of iodine stores and intake in adults and children [13], but data on Tg levels in pregnancy are limited and inconsistent [14,15]. In a recent study on iodine supplementation involving three groups of women (euthyroid pregnant women not given iodine supplements; women given iodine supplements prior to conception; and women given iodine supplements at the beginning of their pregnancies), UIC differed considerably between the women with and without iodine supplementation, reflecting the ongoing supplementation, while Tg dropped only in women with longstanding iodine supplementation, reflecting pre-existing iodine thyroid stores. Therefore, Tg at mid-pregnancy was the most reliable marker of iodine status before conception and in the first trimester of pregnancy—the most crucial period for fetal brain development. [16].

In the light of the conflicting data, the American and European Thyroid Associations both recommend iodine supplementation (150 ug/day) in pregnancy, whereas WHO does not recommend it for women living in countries running effective and sustained iodized salt programs. In other words, it is still not clear whether iodine supplementation is warranted during pregnancy in developed areas where iodized salt is available [17]. From 2005, Italy has had a sustained iodized salt program, although it is still sub-optimally effective on a national level, since the iodized salt coverage is around 65% of households [18]. We recently documented a more widespread use of iodized salt in our region, however, reaching 84% of the population [19]. While data collection on iodine status in pregnant women is still ongoing nation-wide in our country, a previous study by our group on a large number of pregnant women residing in our region documented a median UIC of 83 ug/L [20]. On that basis, pregnant women residing in the Veneto Region are considered to have mild-to-moderate iodine deficiency. According to WHO recommendations and a recent Italian Consensus, gynecologists are only advised to suggest the use of iodized salt and foods rich in iodine [17,21].

Our study was conducted in an area of mild-to-moderate iodine deficiency with a sustained iodine prophylaxis program based on iodized salt, aiming to assess: (a) changes in UI/Creat induced by iodine supplementation during pregnancy and breastfeeding; (b) the relationship between iodine supplementation and thyroid function; (c) and differences in Tg levels and thyroid volume (TV) between women with and without iodine supplementation during pregnancy.

## 2. Materials and Methods

We conducted a single-centered, randomized, single-blinded and placebo-controlled trial. The tablets used (iodine and placebo) were identical in appearance and flavor. The study was performed in accordance with the guidelines of the Helsinki Declaration, and approved by the Local Ethical Committee (Padua General Hospital, code number 0035273), and all patients gave their written informed consent. The 3:2 randomization was chosen [22].

We enrolled women with a singleton pregnancy who presented for prenatal care before 12 weeks of gestation in Padua and the surrounding province. Women more than 16 years old, and residents in Italy, met our inclusion criteria. Our exclusion criteria concerned twin pregnancy, any personal history of autoimmune diseases, treatment with L-T4, intestinal malabsorption, and refusal of informed consent. The recruitment period lasted 14 months (from September 2016 to November 2017) and the observation period ended in September 2018. Ninety pregnant women were enrolled—65 Italians and 25 foreigners (mainly from Eastern Europe).

An alpha-numerical code associated with the type of treatment was consecutively and sequentially assigned to each woman at the time of their enrollment. Among the 90 women enrolled, 52 were given the iodine supplements (225 ug/day) in the form of potassium iodide tablets (Ibsa, Italy), and 38 were given the placebo (Ibsa, Italy) up until 8 weeks after delivery. The study was divided into 4 phases: recruitment (T0) (before the 12th week of gestation), second trimester (T1) (17th–25th week of gestation), third trimester (T2) (29th–38th week of gestation), and post-partum (T3) (8 weeks after delivery).

At each visit, patients provided an early-morning spot urine sample to assay urinary iodine and creatinine concentration (the UI/Creat ratio was used to measure iodine status), and a blood sample to measure TSH, FT4, FT3, and Tg. During the last evaluation, a urinary sample was obtained from the newborn to measure UIC in 16 cases. The newborns’ TSH levels were obtained in all cases from the congenital hypothyroidism screening program.

Thyroid ultrasound was performed (EUB 7500, Hitachi Medical Corporation 4-14-1, Soto-kanda, Chiyoda, Tokyo, Japan) with a 7.5 mHz linear electronic transducer to assess thyroid volume, echogenicity, and vascularization. The parameters considered for both lobes were longitudinal diameter (Ld), transversal diameter (Td), and anteroposterior diameter (APd). The following formula was used to estimate the volume: TV (mL) = Ld × Td × APd × π/6 (right lobe) + Ld × Td × APd × π/6 (left lobe). A TV > 18 mL was considered higher than normal [23]. Maternal weight and height were also recorded at the baseline.

At T0, T2, and T3, the women were administered a food frequency questionnaire regarding their use of iodized salt at home (and for how many years it had been used), and their daily dietary intake of cow’s milk, yogurt, cheese, meat, eggs, fish, and soymilk. Milk intake was scored as: 1 = none; 2 = occasional consumption; 3 = one cup a day (at least 200 mL/day); and 4 = more than 1 cup a day. For other foods (i.e., yogurt, cheese, meat, eggs, fish, etc.), intake was scored as: 1 = never; 2 = at least once a week; 3 = more than once a week. The women’s demographic data recorded for the purposes of the study concerned ethnicity, country of origin, and years of residency in Italy. The women were also asked what they knew about the iodine prophylaxis program, and the importance of dietary iodine supplementation was stressed at the time of their recruitment.

### 2.1. Laboratory Assays

UIC was expressed as ug/L and measured as described elsewhere [24]. Serum TSH, FT3, and FT4 levels were tested with commercial kits (Roche, Rotkreuz, Switzerland), considering as the reference range: TSH: 0.2–4 mIU/L; FT3: 3.90–6.80 pmol/L; FT4: 9.00–22.00 pmol/L. Tg was measured with a chemiluminescent immunoassay (CLIA; Beckman Coulter, USA), the normal range being from 0 to 50 ng/mL. Urinary creatinine was measured with an enzymatic method (Roche Cobas 8000 Modular Analyzer, IN, USA), reference range: 0.1–54 mmol/L.

### 2.2. Statistical Analysis

The Kolmogorov-Smirnov test was used to test the normal distribution of UI/Creat. As the values were not distributed normally; they are reported here as medians and 95% confidence intervals (95%CI). The same procedure was applied to TSH, FT4, FT3, Tg, TV, and newborns’ TSH levels. The Mann–Whitney test was used to correlate UI/Creat, Tg, TSH, FT4, and FT3 with the two (‘Iodine’ and ‘Placebo’) groups, and to correlate UI/Creat dichotomized for 150 ug/g with Tg and TSH. Correlation tests were used to verify the relationship between BMI and TV, and between UI/Creat and Tg. A covariance test was used to analyze the trends of UI/Creat, TSH and Tg throughout the study. Wilcoxon’s analysis was used to test for differences in TV and in Tg at T0 versus T1, and at T0 versus T2 within the same groups. A *p* value of <0.05 was considered statistically significant.

## 3. Results

Of the 90 women included in the study, 64/90 (71%) were Italian, and 26/90 (29%) were of foreign origin (18 Caucasian, three African, three South American, and three Asian). Their median age at recruitment was 31 years (range: 20–41). Iodine and placebo were distributed with a 3:2 randomization to 52 women in the Iodine group, and 38 in the Placebo group. There were 21 dropouts between T1 and T3, 14/52 in the iodine-treated group, and 7/38 in the placebo-treated group. Dropout occurred at T2 and T3: many foreign women went back to their country of origin to deliver or after delivery; other women had problems coming to our center in the late phases of pregnancy and after delivery; many others stopped taking the tablets or started taking multivitamins (also containing iodine) and were excluded from the study.

At recruitment, the Iodine and Placebo groups did not show any substantial differences in the main anthropometric, educational, thyroid morphological, or functional parameters (Table 1).

Median UI/Creat at recruitment was 53.3 ug/g (IC 95% 43.72–67.20). At T0, in the whole series, UI/Creat was <50 ug/g in 43/90 women (47.8%), ≥50 ug/g but <149 ug/g in 41/90 (45.6%), ≥150 ug/g but <249 ug/g in 5/90 (5.6%), and ≥250 ug/g in 1/90 (1.1%).

Considering UIC without correcting for creatinine values, according to WHO standards, the median UIC in our series was 56 ug/L (IC 95% 30.8–65.9) at recruitment. At T0, it was <20 ug/L in 0/90 women (0%), ≥20 ug/L but <50 ug/L in 42/90 (47%), ≥50 ug/L but <149 ug/L in 31/90 (34%), ≥150 ug/L but <249 ug/L in 15/90 (17%) and ≥250 ug/L in 2/90 (2%).

**Figure 1:**
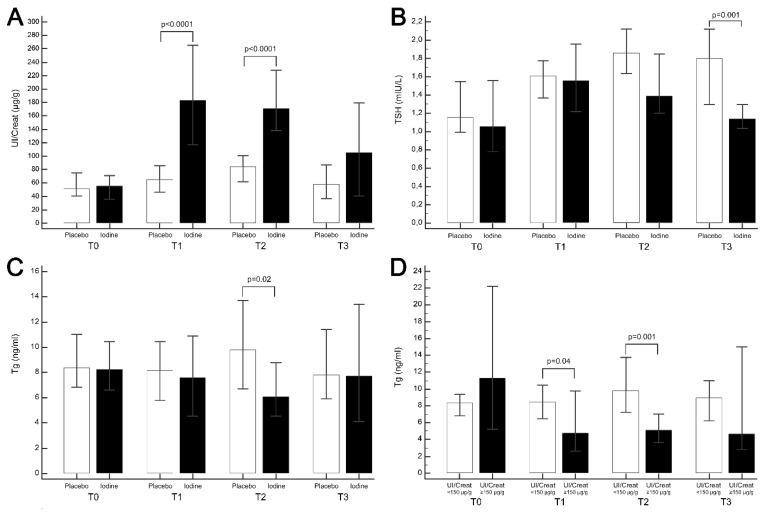
(**A**) Median urinary iodine-to-creatinine concentrations (UI/Creat, ug/g) in the Iodine and Placebo groups at recruitment (T0), in the second trimester (T1), in the third trimester (T2), and 8 weeks post-partum (T3); (**B**) median TSH values (mIU/L) in the Iodine and Placebo groups at T0, T1, T2, and T3; (**C**) median thyroglobulin (Tg) values in the Iodine and Placebo groups at T0, T1, T2, and T3; (**D**) median Tg levels in women with a UI/Creat <150 ug/g and ≥150 ug/g.

Significant differences in UI/Creat emerged between the Iodine and Placebo groups at T1 (with median UI/Creat 183.23 and 65.54 ug/g, respectively; *p* < 0.0001), and at T2 (median UI/Creat 171.16 ug/g and 84.19 ug/g, respectively; *p* < 0.0001), but not at T3, though it was still higher in the Iodine group (median UI/Creat 104.88 ug/g and 58.24 ug/g, respectively; *p* = 0.10) (Figure 1A).

As regards cow’s milk consumption, 21/90 women (22%) drank none, 31/90 (34%) drank it occasionally, 37/90 (41%) drank 1 cup (200 mL) a day, and 1/90 (1%) drank more. Although not significantly, a greater cow’s milk consumption correlated with a higher UI/Creat. Iodized milk consumption was also not significantly related to a higher UI/Creat at the time of recruitment.

Nineteen of the 90 women (21%) knew about the iodine prophylaxis program, and there was no correlation between their knowledge of it and their education or nationality. There was also no correlation between education and UI/Creat at the baseline.

The median TSH in our sample as a whole was 1.12 mIU/L (95%CI 0.96–1.35). TSH did not differ significantly between the Iodine and Placebo groups at T0: it was 1.06 mIU/L in the Iodine group, and 1.16 mIU/L in the Placebo group (*p* = 0.36). There was a significant difference between the Iodine and Placebo groups in the trend of TSH during the course of the study (*p* = 0.02), upon analysis of the covariance; and at T3, it was significantly lower in the Iodine group (1.14 mIUI/L) than in the Placebo group (1.8 mIU/L; *p* = 0.001) (Figure 1B). No significant differences emerged at any of the time points when TSH levels were correlated with UI/Creat </≥ 150 ug/g.

**Figure 2:**
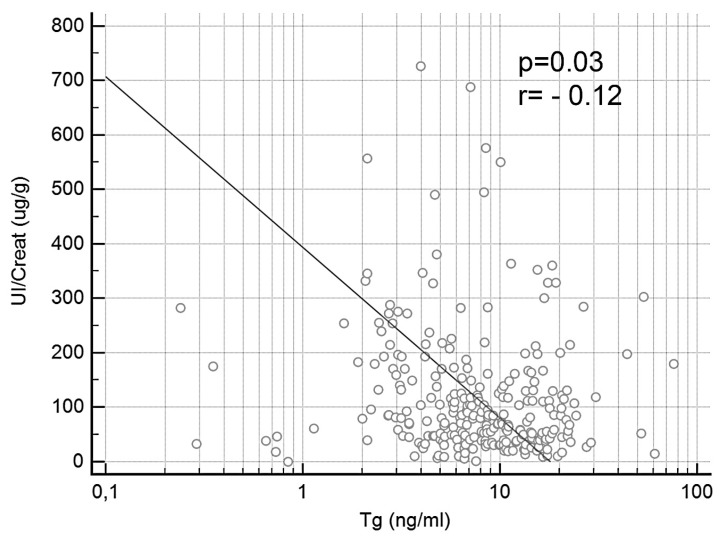
Correlation between iodine/creatinine ratio and thyroglobulin.

When FT4 and FT3 levels were analyzed, the median values in the whole sample were 14.94 pmol/L (95%CI 14.51–15.29) and 4.71 pmol/L (95%CI 4.61–4.97), respectively. There was no significant difference between the two groups’ median FT4 and FT3 levels at T0. Median FT4 was 14.91 pmol/L in the Iodine group, and 15.08 pmol/L in the Placebo group (*p* = 0.27); and median FT3 was 4.71 pmol/L in the Iodine group, and 4.75 pmol/L in the Placebo group (*p* = 0.63). FT4 and FT3 levels did not differ between the two groups at T1, T2, or T3, either. Median Tg in the whole sample was 8.36 ng/mL (95%CI 6.81–9.61). Tg levels did not differ between the two groups at T0, with 8.2 ng/mL in the Iodine group and 8.36 ng/mL in the Placebo group. Statistically significant differences emerged when the trend of Tg was compared between the two groups (*p* = 0.007), and in the Tg levels at T2, when they were 6.07 ng/mL in the Iodine group, and 9.80 ng/mL in the Placebo group (*p* = 0.02) (Figure 1C). A UI/Creat ≥150 ug/g was associated with significantly lower Tg levels: at T1, median Tg levels were 8.47 ng/mL in women with UI/Creat <150 ug/g, as opposed to 4.76 ng/mL in those with UI/Creat ≥150 ug/g (*p* = 0.04); and at T2 (9.8 ng/mL in the former versus 5.08 ng/mL in the latter; *p*= 0.001); but not at T3, although the Tg levels were still lower in patients with a higher UI/Creat (median 8.95 ng/mL in women with UI/Creat <150 ug/g versus 4.63 ng/mL in those with UI/Creat ≥150 ug/g; *p* = 0.31) (Figure 1D). Using Wilcoxon’s analysis on the Iodine and Placebo groups, a significant reduction in Tg levels was documented, starting from the second trimester (*p* = 0.008) and going into the third trimester (*p* = 0.01) in the Iodine group, but not in the Placebo group. There was also a significant negative correlation between Tg levels and UI/Creat at T1 (*p* = 0.01, *r* = −0.31), and T2 (*p* = 0.01, *r* = −0.29), but not at T3, when the negative correlation remained, but was no longer significant (*p* = 0.48, *r* = −0.13). On the whole, UI/Creat and Tg were negatively correlated throughout the study (*p* = 0.03, *r* = −0.12) (Figure 2).

Median TV in the whole sample was 10491.51 mm^3^ (95%CI 9683.73–12022.08). It did not differ between the Iodine and Placebo groups at the baseline, at which point the median TV values were 10823.80 mm^3^ (95%CI 9813.94–12588.26) and 9701.12 mm^3^ (95%CI 9208.77–12181.21), respectively. A significant correlation emerged between TV and BMI (*p* = 0.01, *r* = 0.3653). There was no statistically significant difference in median TV between the Iodine and Placebo groups at T1 (11222.64 mm^3^ and 10592.40 mm^3^, respectively), at T2 (11550.24 mm^3^ and 10743.20 mm^3^, respectively), or at T3 (11662.04 mm^3^ and 9453.60 mm^3^, respectively). On the other hand, the Placebo group’s median TV increased significantly from T0 (9552.14 mm^3^) to the second trimester (10696.92 mm^3^; *p* = 0.007), and continued to increase in the third (to 10862.54 mm^3^; *p* = 0.0009), whereas the Iodine group’s median TV only increased significantly (by a marginal significance) in the third trimester (12368.46 mm^3^, as opposed to 11450.14 mm^3^ at T0; *p* = 0.03). The percentage variation in TV from T0 to T2 was +Δ7.43% in the Iodine group, and +Δ11.17% in the Placebo group. The significance of the increase in TV was lost post-partum.

UIC was analyzed in only 16 newborns (due to mothers’ urine sampling difficulties)—nine in the Iodine group, and seven in the Placebo group. It was significantly higher in the offspring of women in the Iodine group (302.89 ug/L versus 92.00 ug/L for the Placebo group, *p* = 0.01).

All the results regarding the comparison between the Iodine and Placebo groups are summarized in Table 2.

At screening, no differences in TSH were documented between newborns in the two groups, their median TSH being 2.7 mIU/L (95%CI 2.4–3.3 mIU/L) in the Iodine group and 3.6 mIU/L (95%CI 2.4–5.3) in the Placebo group.

Three miscarriages occurred in our sample, one in the Iodine group, and two in the Placebo group (*p* = ns).

## 4. Discussion

To our knowledge, this is the first randomized and placebo-controlled trial to have been conducted in an area of mild-to-moderate iodine deficiency with a sustained iodine prophylaxis program based on iodized salt, in which UI/Creat and thyroid function parameters, including Tg, were measured in all three trimesters of pregnancy and post-partum, during which time UI/Creat was adequate in the iodine-supplemented intervention arm compared with that of the iodine-deficient group given the placebo [4,25]. It is worth noting that women were recruited (and given any supplements) right from the first trimester.

Our sample of women had a median UI/Creat in the range of mild-to-moderate iodine deficiency at the time of their recruitment. The median UI/Creat obtained at recruitment in the present series was lower than expected in the light of previous data obtained in a large number of consecutive pregnant women residing in our region [20], but still consistent with a mild-to-moderate iodine deficiency. Consistently with this assumption, Tg values at recruitment were in the range of mild-to-moderate iodine deficiency. Despite running education programs about iodine, the Veneto region (in north-eastern Italy) is therefore, presumably still an area of iodine deficiency, in girls and women of childbearing age at least, as recently shown by our group [26]. In fact, our Placebo group also retained the same iodine levels throughout the study, unlike the case of other intervention studies conducted in areas of mild-to-moderate iodine deficiency [27]. At the time of their recruitment, no correlations emerged between our women’s iodized salt and cow’s milk consumption and their iodine sufficiency. This could be because their mean daily consumption of 200 mL of cow’s milk is not enough to reach the iodine levels recommended in pregnancy. Educational intervention on the use of iodized salt and dietary compounds rich in iodine prompted a slight but statistically insignificant median increase in UI/Creat in our Placebo group. In other words, confirming reports from other groups [28], for pregnant women living in areas of genuine mild-to-moderate iodine deficiency, educational intervention is not enough to ensure an adequate iodine intake. Iodine supplementation in our Iodine group sufficed to raise the women’s median UI/Creat adequately (to 150–249 ug/g), having done so already at the second trimester and up until the end of the pregnancy. Their median UI/Creat fell to below 150 ug/g in the post-partum period, a finding also reported in another study [27] that may be due to the return to lower renal filtration rates after delivery [29], and to the transfer of iodine to the newborn by lactation. This is supported by our data, since the median UIC in the newborns of the Iodine group was higher than the one of the Placebo group.

Tg had previously proven a promising functional biomarker of iodine status during pregnancy (performing better than TSH) in a cohort of mildly-to-moderately iodine-deficient women in the United Kingdom [14]. We confirmed that Tg levels rose as of the second, and especially the third trimesters in our Placebo group; conversely, a similar Tg increase trend was not observed in the Iodine group, where Tg values became significantly lower by comparison with the Placebo arm at the third trimester. Moreover, Tg values were significantly lower in women with a UI/Creat ≥150 ug/g in the second and third trimesters. A negative linear correlation between UI/Creat and Tg confirmed this finding. In the first trimester, Tg did not correlate with iodine intake, possibly because of the powerfully stimulating action of chorionic gonadotropin (hCG) on the TSH receptor during this period, and the consequent increase in thyroid hormone and Tg production, as previously stated [15].

Our data suggest that Tg could be a good marker of iodine sufficiency in mid–late pregnancy, and the better correlation found in the third trimester suggests that it particularly reflects long-term iodine intake rather than recent intake [14,16,30]. Our randomized placebo-controlled study correlated Tg and UI/Creat in all three trimesters of pregnancy and post-partum in a population of mildly-to-moderately iodine-deficient pregnant women, some of them in a supplemented iodine-sufficient group. Previous studies in iodine-deficient areas showed that Tg values were higher in pregnant than in non-pregnant control women, due to increased thyroid stimulation to preserve euthyroidism, while no increase in Tg levels was recorded in the case of normal iodine availability [31,32]. Moreover, several studies also found a correlation between Tg and UIC during pregnancy in areas of iodine deficiency [6,33,34], whereas such a correlation is lost in other settings, both in iodine-sufficient and in iodine-deficient scenarios [31,33]. The iodine abundance of thyroid stores before pregnancy plays an important part, being a possible source of iodine even in the case of suboptimal iodine supply during gestation [33]. The higher median Tg levels seen in our Placebo group suggest thyroid hyperstimulation, reflecting the greater increase in TV seen in conditions of low iodine intake. Even in an area of only mild-to-moderate iodine deficiency like ours, iodine supplementation supported thyroid function during pregnancy—although the consequences for the mother and fetus of the thyroid hyperstimulation, as seen in our non-supplemented women, are still unknown.

TSH, FT4, and FT3 proved unsuitable as markers of iodine status in our women. This may be partly because they come under strict homeostatic regulation [30], and partly due to laboratory pitfalls during pregnancy [2]. Indeed, we feel that the key finding is represented by different TSH trends in the two groups: TSH dropped increasingly throughout the pregnancy in the Iodine group, unlike the non-supplemented arm, and it was also significantly lower in the post-partum period. We speculate that this last result could be due to the different homeostatic conditions and thyroid economy in the post-partum period in comparison to gestation [35,36], or could be due to the slow response of TSH to physiological and non-physiological changes.

Our data, obtained in a placebo-controlled prospective setting, suggest that iodine supplementation is not harmful for mildly-to-moderately iodine-deficient pregnant women, and has no appreciable detrimental effect on the thyroid gland or thyroid hormone production—a cause of concern in various studies [16,28,37]. A previous study in the same setting found lower FT4 levels in the third trimester in iodine-supplemented pregnant women, but they were not in the range of mild-to-moderate deficiency before starting iodine supplementation [27]. Our results come from a relatively small study population and the absence of a detrimental effect on maternal thyroid function would need to be confirmed in a larger series. The newborn’s neurological function was not investigated in our series, and this also limits the value of our findings. To confirm the safety of the intervention, larger RCT and placebo-controlled trials need to be conducted in areas of mild-to-moderate iodine deficiency like the Veneto region, and children’s neurodevelopment should be included among the aims of the study.

The albeit limited data on the newborns’ UICs in our study showed very high levels in the iodine-supplemented group. This finding is unreliable, however, because of the small size of the sample tested.

Based on our findings, iodine supplementation during pregnancy did not modify TSH values in newborns at screening, as previously observed [27].

Our study has several weaknesses. The high rate of dropouts and the rather small final number of women partially limit the value of the results obtained. Moreover, although UI/Creat reflects 24-h urinary iodine excretion better than UIC [12], it is still only a single measure of iodine status and possibly not representative of an individual’s real iodine intake.

Judging from our data, iodine supplementation in areas of mild-to-moderate iodine deficiency can prevent the thyroid hyperstimulation caused by the increase in iodine demand during pregnancy without giving rise to any thyroid dysfunction. The trend of Tg during pregnancy is a good indicator of whether an adequate iodine intake has been achieved, mainly as of the second trimester and particularly in the third.

In conclusion, we demonstrated that iodine supplementation of 225 ug/day in pregnant women from the first trimester onwards is not detrimental and that it helps to prevent thyroid hyperstimulation, possibly also avoiding the possible detrimental impact of mild-to-moderate iodine deficiency on fetal neurodevelopment [7,8,38]. Iodine supplementation during pregnancy is not the rule in healthy pregnant women in Italy today, especially not for foreign women. Although data on fetal neurodevelopment were not available for our series, we demonstrated that women not receiving an iodine supplement remained iodine-deficient throughout their pregnancies, in spite of medical advice to use iodized salt and iodine-rich foods. We, consequently, certainly cannot rule out the possibility that this deficit could profoundly affect their children’s future cognitive performances. In the light of our findings, it is now time to extend iodine supplementation to all healthy pregnant women in our country too.

## Figures and Tables

**Table 1:** Patients’ characteristics at the baseline.

Parameter	Iodine Group		Placebo Group		*p*
	N = 52	Median (IC95%)	N = 38	Median (IC95%)	
Age (years)		31 (IC95% 29–33)		33 (IC95% 31–33.5)	0.23
Italian nationality		36/64 (56%)		28/64 (44%)	0.65
BMI (kg/m2)		22.0		20.5 (IC95% 19.0–22.0)	0.24
		(IC95% 20.59–22.41)			
Gestational weeks at recruitment		11 (IC95% 10–12)		10 (IC95% 9.5–11)	0.05
Education					
• Bachelor		30/52 (58%)		20/36 (56%)	0.84
• Degree		22/52 (42%)		16/36 (44%)	“
Cow’s milk consumption					
• no/rarely		30/52 (58%)		22/38 (58%)	0.98
• daily		22/52 (42%)		16/38 (42%)	“
Iodized salt		50/52 (96%)		35/38 (92%)	0.18
UI/Creat (µg/g)		55.37		50.98	
		(IC95% 35.39–71.07)		(IC95% 40.39–75.04)	0.66
		10823.80		9701.12	
Thyroid volume (mm3)		(IC95% 9813.94–12588.26)		(IC95% 9208.78–12181.21)	0.25
TSH (mIU/L)		1.06		1.16	0.37
		(IC95% 0.78–1.56)		(IC95% 0.99–1.55)	
Tg (ng/mL)		8.3		8.36	0.78
		(IC95% 6.62–10.45)		(IC95% 6.82–11.01)	
fT4 (pmol/L)		14.91		15.08	0.27
		(IC95%14.21–15.25)		(IC95%14.34–15.89)	
fT3 (pmol/L)		4.71		4.75	0.63
		(IC95% 4.60–4.98)		(IC95% 4.59–5.09)	

**Table 2:** Maternal laboratory test findings during pregnancy, at the baseline, in the second and third trimesters, and post-partum.

		Baseline		Second Trimester			Third Trimester			Post-Partum		
		N	Median (IC95%)	N	Median (IC95%)	*p*	N	Median (IC95%)	*p*	N	Median (IC95%)	*p*
UI/Creat (ug/g)	Placebo	38	55.37 (35.39–71.07)	38	65.55 (46.62–86.00)	<0.0001	33	84.19 (61.86 to 100.61)	<0.0001	31	58.24 (36.63 to 87.09)	0.10
	Iodine	52	50.98 (40.39–75.04)	50	183.23 (117.39–264.92)		40	171.16 (138.05–228.46)		38	104.88 (40.70 to 179.66)	
Tg (ng/mL)	Placebo	38	8.3 (6.62–10.45)	38	8.16 (5.79–10.44)	0.56	33	9.8 (6.69–14.29)	0.02	31	7.79 (5.90–11.41)	0.67
	Iodine	52	8.36 (6.82–11.01)	50	7.56 (4.53–10.89)		40	6.07 (4.52–8.78)		38	7.67 (4.11 to 13.42)	
Thyroid volume (mm^3^)	Placebo	38	9701.12 (9208.78–12181.21)	38	10592.40 (8563.97–12142.60)	0.11	33	10743.20 (10136.81–11943.49)	0.31	31		0.20
	Iodine	52	10823.80 (9208.78–12181.21)	50	11222.64 (9801.17–14952.06)		40	11550.24 (10232.54–13237.92)		38	11662.04 (9168.88–13099.32)	
TSH (mIU/L)	Placebo	38	1.16 (0.99–1.55)	38	1.60 (1.37–1.78)	1.00	33	1.86 (1.64–2.12)	0.09	31	1.80 (1.29–2.12)	0.001
	Iodine	52	1,05 (0.78–1156)	50	1.55 (1.22–1.96)		40	1.39 (1.19–1.85)		38	1.14 (1.04–1.29)	
FT4 (pmol/L)	Placebo	38	15.08 (14.34–15.98)	38	12.94 (12.41–13.57)	0.52	33	13.04 (12.60–13.34)	0.83	31	13.45 (12.91–13.99)	0.48
	Iodine	52	14.91 (14.20–15.25)	50	12.82 (11.99–13.62)		40	12.66 (12.08–13.52)		38	13.08 (12.42–14.06)	
FT3 (pmol/L)	Placebo	38	4.75 (4.59–5.09)	38	4.06 (3.96–4.27)	0.18	33	4.33 (4.04–4.47)	0.86	31	4,4450 (4.19–4.69)	0.12
	Iodine	52	4.71 (4.60–4.99)	50	4.33 (4.17–4.47)		40	4.21 (4.07–4.43)		38	4.27 (4.08–4.39)	
